# Coronal joint spaces of the Temporomandibular joint: Systematic review and meta-analysis

**DOI:** 10.4317/jced.52439

**Published:** 2015-07-01

**Authors:** Eugénio Martins, Joana-Cristina Silva, Carlos A. Pires, Maria-João-Feio Ponces-Ramalhão, Jorge-Dias Lopes

**Affiliations:** 1 Rua Dr. Manuel Pereira da Silva, 4200-393 Porto, Portugal. Department of Orthodontics, Faculty of Dental Medicine, University of Porto; 2 Department of Orthodontics, Faculty of Dental Medicine, University of Porto; 3 Departamento de matemática, Universidade de Trás-os-montes e Alto Douro

## Abstract

**Introduction:**

The joint space measurements of the temporomandibular joint have been used to determine the condyle position variation. Therefore, the aim of this study is to perform a systematic review and meta-analysis on the coronal joint spaces measurements of the temporomandibular joint.

**Material and Methods:**

An electronic database search was performed with the terms “condylar position”; “joint space”AND”TMJ”. Inclusionary criteria included: tomographic 3D imaging of the TMJ, presentation of at least two joint space measurements on the coronal plane. Exclusionary criteria were: mandibular fractures, animal studies, surgery, presence of genetic or chronic diseases, case reports, opinion or debate articles or unpublished material. The risk of bias of each study was judged as high, moderate or low according to the “Cochrane risk of bias tool”. The values used in the meta-analysis were the medial, superior and lateral joint space measurements and their differences between the right and left joint.

**Results:**

From the initial search 2706 articles were retrieved. After excluding the duplicates and all the studies that did not match the eligibility criteria 4 articles classified for final review. All the retrieved articles were judged as low level of evidence. All of the reviewed studies were included in the meta-analysis concluding that the mean coronal joint space values were: medial joint space 2.94 mm, superior 2.55 mm and lateral 2.16 mm.

**Conclusions:**

the analysis also showed high levels of heterogeneity. Right and left comparison did not show statistically significant differences.

** Key words:**Temporomandibular joint, systematic review, meta-analysis.

## Introduction

One of the main components of the TMJ is the mandibular condyle as it connects the mandible, the only bone of the craniomandibular complex that moves, to the temporal bone by the TMJ. Therefore, the mandibular condyle position has been advocated by several authors to be a main factor of equilibrium of the masticatory system and its ideal position has been a very controversial issue during the past years.

Several hypotheses have been proposed from the most retruded position of the condyle in the glenoid fossa to the most superior, to the current most anterosuperior position with the disk in between (1-3). In the meantime, the relationship between changes in condylar position and the presence of temporomandibular disorders (TMD) is also very controversial within the scientific community (4-7).

As there is some evidence suggesting the influence of dental occlusion on the mandibular condyle position, it is easily understood the importance of determining the condyle position to perform complex rehabilitations and orthodontic treatments (6). According to Hidaka *et al.* (8) 38,7% of orthodontic patients suffer of a degree of condylar displacement that may jeopardize the treatment plan (8). Therefore, it becomes very clear the importance of including the determination of condyle position during orthodontic diagnostic procedures.

There are several methods described in the literature to determine condylar position, including radiographic techniques (9-12). Although, only with the introduction of the evaluation of the TMJ in Laminographies suggested by Robert Ricketts, it became possible to radiographically quantify the joint space measurements and determine the condyle position (9).

Since then, the evolution of radiology has allowed to perform three-dimensional analysis of the structures and accurately determine several measurements, including TMJ spaces on computed tomography (CT), cone-beam computed tomography (CBCT) and magnetic resonance imaging (MRI) (13-18). Many studies have been performed to determine condyle position, both on the sagittal and coronal plane, using mainly CT and CBCT as these exams are more common in dental practice.

The aim of this study is to perform a systematic review of the literature and meta-analysis concerning the coronal joint spaces to define the ideal coronal joint spaces.

## Material and Methods

-Information sources and search strategy

A comprehensive electronic database search to identify relevant publications was conducted, and the reference lists in relevant articles were searched manually for additional literature. No language restrictions were set although no attempt to explore the informally published literature was made. The following databases were searched: Medline (Pubmed), Lilacs, Scopus, Ebsco (Host by University of Porto), Cochrane Central Register of Controlled Clinical Trials.

A search was performed with the terms “condylar position”; “joint space”AND”TMJ” with no year of publication restriction in order to include the highest number of articles (to 22 April 2014). No restriction to study design was applied.

Faculty of Dental Medicine of University of Porto and Portuguese Society of Dentofacial Orthopedics’ libraries were also consulted for printed articles not available online.

-Selection criteria

At the first stage, two reviewers independently screened the titles of the retrieved records, and only the titles related to temporo-mandibular joint spaces were included. Next, the abstracts of the retrieved publications were read by the two reviewers and categorised according to the method used to determine condylar position. An article had only to be justified by one reviewer to be included in the second selection phase. Eligibility of the retrieved articles was determined by applying the following inclusion criteria: (1) tomographic examination of the TMJ (2) determination of coronal joint space measurements at least on two different points.

The main reasons for exclusion were: mandible fractures, studies not performed in living humans, surgical interventions, studies with patients with syndromes or chronic diseases (including degenerative pathology of the TMJ), examination of the condylar position only with clinical methods, 2D radiographs or magnetic resonance imaging, orthodontic or splint therapy, samples containing only patients in the primary or mixed/ early permanent dentition, case reports, discussion or debate articles. All not published studies were also excluded.

The analysis was based on primary materials. When an abstract was considered by at least one author to be relevant, it was read in full text. At the second stage, the full texts were retrieved and critically examined. Reference lists from the articles selected on the second stage were screened and articles related to condylar position assessment by joint space measurements were hand-searched. Book chapters and reviews were excluded since the aim of this systematic review was to evaluate primary studies.

-Data treatment

The following data were extracted from the selected articles: year of publication, study type, study method, sample description, joint space measurements on the coronal plane, error analysis method, statistical analysis and author’s conclusion. One reviewer author then extracted the mentioned data from the included articles and the second author checked. Any disagreement was resolved with discussion between the two authors until a consensus was reached. The risk of bias was assessed according to the “Cochrane risk of bias tool” (19) as suggested by the “PRISMA statement for reporting systematic reviews and meta-analyses of studies that evaluate health care interventions: explanation and elaboration” (20).

-Meta-analysis

The values studied in this meta-analysis were the coronal joint space measurements (medial, superior and lateral joint space) and the differences between the right and left joints. As not all the included articles presented the values for all the spaces from the right and left joints, the analysis were performed including all the data presented in each selected study. For the comparative analysis between the right and left joints, mean and standard deviation values from the samples of each article were used. For global joint space assessment, mean and standard deviation of the total sample (including both the values from the right and left joints) were used.

The restricted maximum-likelihood (REML) method was used to estimate de variability between the studies. Inverse variance method was used to assess the weight of each study (21).

Heterogeneity was determined using the Q Cochran Test and the I² statistics by Higgins and Thompson (21).

Statistical analysis was performed using “R”, version 2.15.2 from “The R Project for Statistical Computing”, available from http://www.r-project.org.

## Results

-Search results

From the initial search strategy 916 articles were retrieved from Medline (Pubmed), 1114 from Scopus, 158 from EBSCOhost, 19 from Lilacs and none from the Cochrane Central Register of Controlled Clinical Trials. The number of articles reviewed in each phase of this systematic review is presented in the PRISMA. After excluding 978 duplicates, 1230 articles remained for review. In the first phase selection, the observers screened the articles by reading titles and abstracts. Articles that were not eligible because of irrelevant aims and were not directly related to this systematic review were excluded, thus 61 articles remained for further reading. 28 articles were assessed for eligibility. After screening all the articles full text according to the inclusion/ exclusion criteria, 4 (10,22-24) articles classified for final review.

-Type of study and method used to determine joint space measurements

No randomized clinical trials (RCTs) have been performed on coronal joint spaces of the TMJ. A prospective study (23) and two retrospective studies (22,24) have been found meeting the eligibility criteria. A prospective and retrospective study was also found (10). Three of the retrieved articles (22-24) performed cone-beam computed tomography (CBCT) to obtain the 3D images of the TMJ, while Christiansen *et al.* (10) used CT images.

Dalili *et al.* (22) measured the distance from the most prominent medial and lateral poles of each condyle to the intersection point of two tangent lines from the deepest point of the glenoid fossa to the respective medial and lateral slopes. In the meantime, Ikeda *et al.* (24) divided the mediolateral width of the condyle in sextants in the coronal view and projected the midpoint perpendicularly to the true horizontal line (THL) to its surface to find the central coronal point. The medial and lateral coronal points derived from lines perpendicular to the THL extending from the junction of the medial or lateral first and second sextants, respectively. The shortest distances from the medial, central and lateral points to the fossa were then measured. At last, Henriques *et al.* (23) identified the most medial and lateral points of the condyle and draw a line and its midpoint was considered to trace another line at 90 degrees and two other at 45 degrees laterally and medially respectively. The intersection point of these lines with the condyle surface and the glenoid fossa were determined and the distance in between measured.

Christiansen *et al.* (10) measured the closest distance between the most centred and superior point of the condyle (CJS) and the most medial point of the condyle (MJS) to the glenoid fossa.

-Quality assessment

The summary of the quality assessment of the reviewed articles is on  Table 1.

**Table 1:**
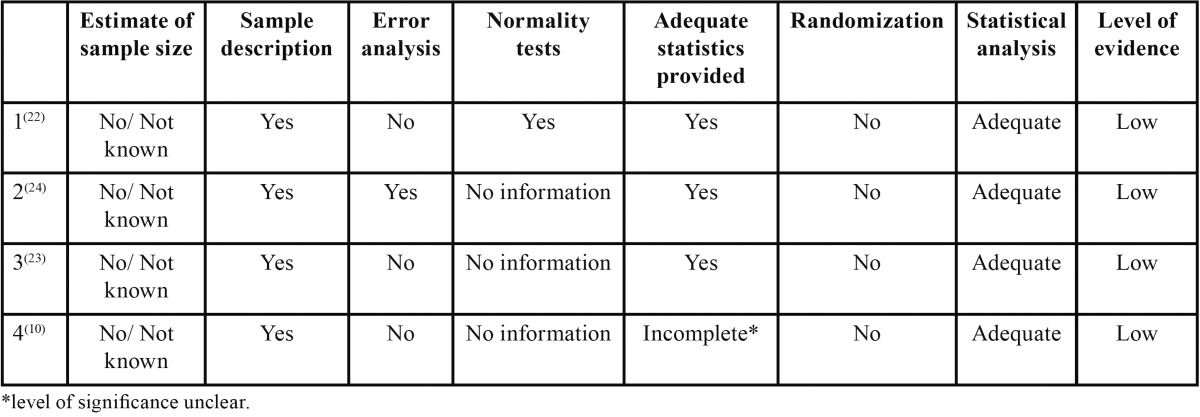
Summary of the quality assessment of the four retrieved articles.

Globally, the statistical analysis performed on each case were adequate to the goals of the research. However, only one article (22) presents normality tests in order to determine the statistical tests to apply. On the other three studies (10,23,24) it is not possible to evaluate the validity of the statistical tests applied (T Student and ANOVA) as they were used in small samples with no information about the normality of the data. One of the selected articles (23) does not present the correlation coefficient used. None of the studies reports estimation of the sample size and method error analysis was only performed on one study (22). In summary, all of the retrieved articles were classified as low level of evidence according to the “Cochrane risk of bias tool”.

-Meta-analysis

The four articles included on the review were used in this meta-analysis. For the medial joint space, the four studies presented the mean values, although the same was not true for the lateral joint space, as Christiansen *et al.* (10) did not measure this space. Similarly, Dalili *et al.* (22) did not consider the superior joint space.

**Figure 1:**
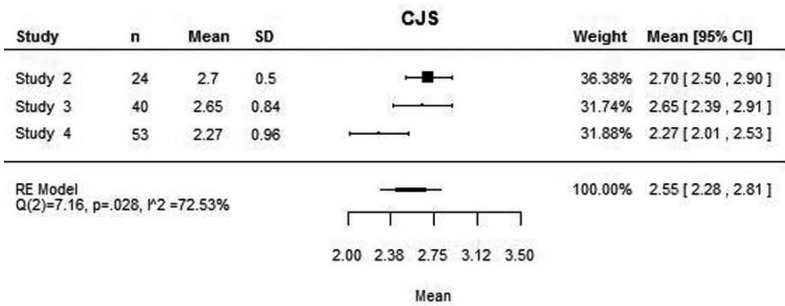
Mean medial joint space value and for each study and mean difference between medial joint space between right and left joint and for each study.

**Figure 2:**
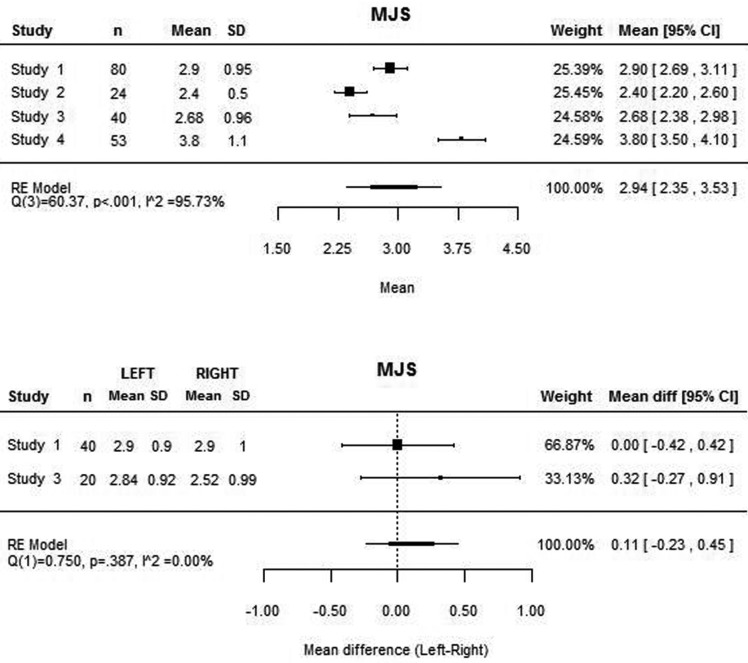
Mean lateral joint space value and for each study and mean difference between lateral joint space between right and left joint and for each study.

**Figure 3:**
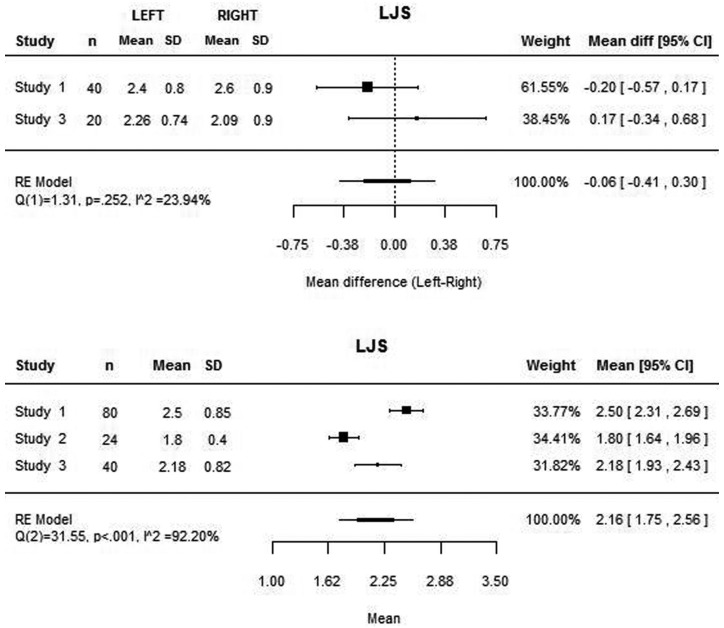
Mean superior joint space value and for each study.

The mean medial, lateral and superior joint space values assessed with this meta-analysis were 2.94 mm, 2.16 mm and 2.55 mm respectively (Figs. 1-3). High heterogeneity was found among the four articles: (Q(3) = 60.37; *P*<0.001; I2 = 95.73%) for the MJS; (Q(2) = 31.55; *P*<0.001; I2 = 92.20%) for the LJS; (Q(2) = 7.16; *P*=0.028; I2 = 72.53%) for the SJS.

The mean differences between the right and left joints are close to zero both to the medial and lateral joint space (Figs. 1,2). However, these values are based only on two studies (22,23) as the remaining do not present the values for each joint separately. Concerning the superior joint space, the values of each joint separately is present only on the study of Henriques *et al.* (23), being the mean difference of 0.35 mm (95% CI: -0.17, 0.87). The heterogeneity analysis shows that the difference values are homogeneous both to the medial and lateral joint space.

## Discussion

Joint space measurements have been used to assess the mandibular condyle position radiographically since Ricketts used this method in laminographies (9). Since then, the technology has evolved so much that it is now possible to assess the joint space in 3D radiographic imaging with CT, CBCT and MRI. Therefore, a systematic review to assess the relevance of these methods and their scientific evidence is necessary. In the present study, all the articles about joint space assessment on 2D radiographic examination of the TMJ were excluded as these methods have proven lower accuracy both in the image acquisition process and in measurements, than 3D radiographic methods (18). MRI was also excluded because this exam is not indicated to assess hard structures and, as both the mandibular condyle and the glenoid fossa that limit the joint space are mainly bone and cartilage, this is not the best exam for accurately determine joint space measurements (25). Furthermore, all the articles including extensive treatment that could significantly influence the joint space, like orthodontic treatment and splint therapy, have been excluded. Finally, studies with samples exclusively on the mixed and early permanent dentition were excluded as the mandibular condyle is not completely formed before the end of the growth, usually between 15 to 16 years old. The exclusion of studies that only assessed the joint space in less than two separate points of the TMJ was due to the definition of the position of an object in space depending on three coordinates. According to this, the analysis of the joint space only on one point does not provide enough information to determine the position of the mandibular condyle in the glenoid fossa.

The review enhanced the lack of studies about coronal TMJ space analysis with tomographic imaging as only four articles matched the eligibility criteria. Furthermore, the retrieved studies present small samples which determine that its results should be read with caution.

As all the studies were classified as low level of evidence according to the “Cochrane risk of bias tool” the authors suggest the need to perform more studies with structured methodology that lead to more solid conclusions.

A meta-analysis of the results of the four retrieved articles was performed. However, the authors are aware that its results should be carefully interpreted as it is based on few studies with low level of evidence.

According to the attained values, the mean MJS, LJS and CJS were 2.94 mm, 2.16 mm and 2.55 mm respectively. However, the analysis also showed high heterogeneity that reduces significantly the power of these values. Therefore, more research is needed in order to achieve more homogeneous values that allow direct comparison of results and solid conclusions.

On the contrary, homogeneity was found among the difference between right and left joint, which suggests the absence of statistically significant differences between both sides. However, this analysis was only based on two studies and should not be considered a strong conclusion.

## Conclusions

The conclusions of this systematic review and meta-analysis concerning the coronal joint space measurements are:

- Lack of scientific evidence, as all the retrieved articles were of low level of evidence;

- The meta-analysis suggest the following mean values for the coronal joint spaces: 2.94 mm MJS, 2.16 mm LJS and 2.55 mm CJS;

- High heterogeneity among the studies;

- Suggestion of the absence of statistically significant differences between the coronal joint space of the right and left joints.
